# The Blood

**Published:** 1888-10-27

**Authors:** 


### Physiology and its Uses.

### THE BLOOD.

The author of the Pentateuch or one of its editors, who certainly wrote more than two, and may have written nearly three, thousand years ago, had the clearest and most definite knowledge of the physiological value of the blood. "The blood is the life," he wrote. It could not, indeed, have been very difficult to discover so much about it as that, because the constant slaughtering of animals and the frequent killing of human beings by means of knives, swords, daggers, and other cutting weapons which caused the blood to escape, would soon render everybody familiar with the sight of creatures first losing consciousness and then dying after the flow of large quantities of blood. This is one of those broad facts of science which are the property of all ages. To the inhabitant of Ancient Egypt and to people of times still earlier the blood was the life; to us also it is still the life; and to our remotest successors we may safely prophesy that this great fact will be the same as it is to us.

Whilst we admit and emphasise the circumstance that this scientific generalisation was familiar to the ancients, we cannot but express profound surprise that the method of the circulation of the blood and the details of its functions and uses were unknown until Harvey's demonstrations. It was more than discreditable to the physicians of many ages that such should have been the case. The depth of their disgrace will appear if we consider the condition of philosophy among the Greeks two thousand years before the time of Harvey, and of religion among the Jewish prophets in still earlier times. Yet more striking was the advanced state of sanitary science among the Hebrews of the desert and of Palestine. It is not only curious, but instructive, to consider what could have been the reason for the delay in discovering such an elementary and apparently obvious fact as that of the circulation of the blood. Was it mere indolence which retarded the progress of knowledge? It was not ignorance alone, but prejudice in a much more marked degree. It would be impossible to believe, if it were not established beyond doubt, that Harvey, in the eyes of many doctors, covered himself with disgrace by the publication of his discovery. To us such crass stupidity, and such apparently wilful and wicked ignorance, as many of the medical men then displayed, seem both ludicrous and worthy of the severest reprobation. But both the ignorance and the prejudice have often been matched in medicine and other arts and sciences. Vaccination excited the profoundest distrust in many minds, and that we cannot wonder at, for it was of the nature of a new departure into fields very remote indeed from every-day experience. Chloroform and its discoverer were alike anathematised with all the energy of professional jealousy and the unreasoning wilfulness of personal stupidity.

There is a purpose to be served in recalling those past unworthinesses of the members of a beneficent and liberal profession. That purpose is to excite the medical men of the present day to honest thought and candour of mind. It is surely unscientific and stupid to the last degree to condemn a discovery or a fresh departure in any art or science merely because it is new. Are medical men so mentally incompetent and so distrustful of their own judgment that they will not look a new thing fairly in the face, but will wait until some other real or supposed authority has pronounced upon it, and then follow that pronouncement blindly at all risks? That is a method fraught with evil consequences, and most of all to those who follow it. The truth is that the medical profession is one in which the exercise of individual freedom is reduced to a minimum. Certain persons claim authority, and often exercise that authority in their own self-interest. That would be a matter of no moment at all, were it not that many medical men are possessed with such an ignoble fear that they will submit to any kind of indignity and meanness of behaviour rather than run the risk of having themselves maliciously spoken of. Such conduct as that is base in itself and highly injurious to the whole profession of medicine. It is not only the right but the duty of every individual medical man to live in the exercise of his own best and honestest judgment. If, in the exercise of that judgment, he comes into collision either with constituted or self-appointed authorities, it is still his duty to follow his honest opinion and take the consequences. Properly-constituted authorities may, indeed, claim a limited power of guidance and control over him, a control, however, which must always stop short when it approaches the region of conscience. Self appointed authorities occupy an entirely different ground, and their interference is to be regarded as an inexcusable impertinence and to be treated with contempt. It is possible to imagine a medical man differing from every other medical man in the world, and yet being in the right. In that case it would be his duty to stand firm, be the consequences what they might. There can never be either true freedom or worthy progress until individual men claim and exercise the rights which God has bestowed, and which noman or assembly of men have any just claim to take away.

Medicine has not had many martyrs. So much the worse for medicine. It is a poor cause which rouses weak convictions and feeble enthusiasms. It has, however, had some, and especially in this country, who have possessed the pluck and unyielding firmness of the true Englishman; and we recall their steadfast determination to mind, in order that others may not be moved, who, like them, are assailed by prejudice, and irrational abuse. That man does not deserve the name of man who yields one inch of reason and conscience to the clamour of unreason or of doubtful expediency.

This digression is not out of place at the commencement of a consideration of the circulation of the blood. There are those who believe, or affect to believe, that it is an improper use of physiological knowledge to make it a means of instruction and education for what they please to call "the laity." This discovery of theirs is quite recent, and is a little late in the day. Professor Huxley, and other distinguished persons, have long recognised the great value of physiological training, both for the important knowledge it imparts and as a means of mental discipline. Acting on their convictions, they have published text books for the use of public and private schools. On lines similar to those laid down in such text books, it is possible to write articles, which, whilst being simple and easy of comprehension, shall yet be perfectly accurate, and, for all practical purposes, complete. Physiology, as we have stated elsewhere, is a study of the profoundest interest. It does not merely concern the bodily health and the arts of the physician, the surgeon, and the pharmacist, it also enters profoundly into many of those questions of life, death, and immortality, which occupy the minds of the metaphysician and the divine. Such a study is emphatically the province of man as man, and not merely of the physiologist as physiologist. He who will in any measure strive to put hindrances and difficulties in the way must be classed with those worthless persons who are too dull and coarse to value knowledge themselves, and too selfish to premit others to acquire it. Such men are as much the enemies of their kind as wild beasts or lawless brigands. It is the misfortune of this country at the present moment that there are branches of the medical press which occupy this unscientific, injurious and entirely retrogade position.

*(To be continued.)*

